# Berberine, A Phytoalkaloid, Inhibits Inflammatory Response Induced by LPS through NF-Kappaβ Pathway: Possible Involvement of the IKKα

**DOI:** 10.3390/molecules26164733

**Published:** 2021-08-05

**Authors:** Kiran Kumar Reddi, Hanxuan Li, Wei Li, Sarada D. Tetali

**Affiliations:** 1 Department of Plant Sciences, University of Hyderabad, Hyderabad TS-500046, India; 2 Present address—Department of Bioscience Research, College of Dentistry, University of Tennessee Health Science Center, Memphis, TN 38163, USA; 3 Department of Pharmaceutical Sciences, College of Pharmacy, The University of Tennessee Health Science Center, Memphis, TN 38163, USA

**Keywords:** berberine, THP-1 cells, lipopolysaccharide, NF-κB, 5TL, anti-inflammation

## Abstract

Berberine (BBR), a plant alkaloid, is known for its therapeutic properties of anticancer, cardioprotective, antidiabetic, hypolipidemic, neuroprotective, and hepatoprotective activities. The present study was to determine the molecular mechanism of BBR’s pharmacological activity in human monocytic (THP-1) cells induced by arachidonic acid (AA) or lipopolysaccharide (LPS). The effect of BBR on AA/LPS activated proinflammatory markers including TNF-α, MCP-1, IL-8 and COX-2 was measured by ELISA or quantitative real-time PCR. Furthermore, the effect of BBR on LPS-induced NF-κB translocation was determined by immunoblotting and confocal microscopy. AA/ LPS-induced TNF-α, MCP-1, IL-6, IL-8, and COX-2 markers were markedly attenuated by BBR treatment in THP-1 cells by inhibiting NF-κB translocation into the nucleus. Molecular modeling studies suggested the direct interaction of BBR to IKKα at its ligand binding site, which led to the inhibition of the LPS-induced NF-κB translocation to the nucleus. Thus, the present study demonstrated the anti-inflammatory potential of BBR via NF-κB in activated monocytes, whose interplay is key in health and in the pathophysiology of atherosclerotic development in blood vessel walls. The present study findings suggest that BBR has the potential for treating various chronic inflammatory disorders.

## 1. Introduction

Berberine (BBR) is an isoquinoline alkaloid, belonging to the class of protoberberine alkaloids, found in many medicinal plants such as *Berberis vulgaris* [1], *Coptidis rhizoma* [2], *Coscinium fenestratum* [3], *Hydrastis canadensis* [4], and *Tinospora cordifolia* [5,6]. BBR-enriched plants have been used in Chinese and Indian traditional medicine to treat diabetes, cardiovascular or neurodegenerative disorders, and other maladies [6]. Accumulated evidence in the literature resulted out of various in vitro and in vivo experimental models document pharmacological activities of BBR, including anticancer [7], cardioprotective [8], antidiabetic [9], hypolipidemic [10], neuroprotective [11], antiviral [12] and hepatoprotective [13] properties. Several clinical studies carried out with BBR have further supported its efficacy against chronic inflammatory diseases such as diabetes [14,15], hypertension [16,17], and hyperlipidemia [18,19]. Recent studies with BBR have shown that it reduces LPS-induced lung injury by attenuating vascular cell adhesion molecule 1 (VCAM-1) in both in vitro (HUVECs) and in vivo (male Sprague Dawley rats) models. Although BBR has been reported to show diverse therapeutic effects, its anti-inflammatory effects on LPS and AA challenged THP-1 cells have not been studied in detail to date. In addition to it, the molecular mechanism of BBR’s inhibitory effect on inflammatory pathway has not been addressed to the best of our knowledge. 

Inflammation is a complicated biological process that develops within the host in response to infection or oxidative stress. It’s occurrence in the blood vessel involves the activation of monocytes and endothelial cells of the vessel wall by releasing certain proinflammatory agents and thus promotes the onset and progression of atherosclerosis [20]. Notionally, one can reduce the risks of complications associated with the atherosclerosis by attenuating the activation of these cell types. LPS is a gram-negative bacterial endotoxin that produces a septic shock upon infection within the body and leads to acute inflammation. LPS migrates into the monocytes by binding to its receptor TLR-4 [20] and activates IKKα/β kinase, which in turn phosphorylates inhibitory molecule IKappaβ (IκB). The phosphorylated IκB undergoes proteosome dependent degradation, freeing NF-κB in the cytosol, which actively translocates into the nucleus [21]. Thus NF-κB, a transcriptional factor, leads to the secretion of proinflammatory cytokines such as TNF-α, IL-6, IL-8, and MCP-1 etc by monocytic cells. These secreted molecules in turn stimulate endothelium [20], enhance the attachment of activated monocytes to endothelium followed by their migration into the blood vessel, leading to the formation of fat laden foam cells considered to be a primary stage leading to atherosclerosis. Arachidonic acid, being an unsaturated fatty acid, activates the phospolipase enzymes in the cell and produces COX-2 enzyme which in turn produces signalling molecules like ROS and peroxides. ROS and peroxides activate the transcription factor Egr1 which enters the nucleus and activates the secretions of various proinflammatory cytokines [22]. IkappaB phosphorylation and NF-kappaB activation are hallmarks of chronic inflammatory diseases, hence forth identifying novel drugs targeting such signaling molecules represent promising therapeutic tools. The objective of the present study is to investigate the effect of BBR on AA/LPS-induced inflammatory response in human monocytic (THP-1) cells and elucidate the molecular mechanism responsible for its inhibitory activities.

## 2. Results

### 2.1. Effect of BBR on Cell Viability of THP-1 Cells

**Figure 1:**
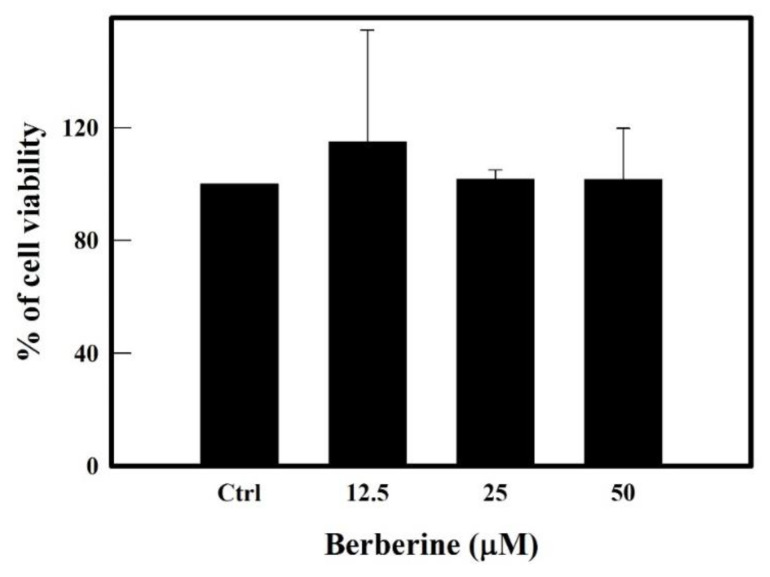
MTT assay of berberine treated THP-1 cells. Cell viability after treating with different concentrations of BBR ranging from 0 to 50 μM for 16 h at 37 °C and 5% CO_2_ was checked by MTT assay. Samples of media alone (without cells) incubated with BBR were used as respective blanks. Ctrl (control) represents cells treated with media alone without berberine. Data represent mean ± SD of three independent experiments.

Results in Figure 1 from MTT assay clearly showed that BBR at tested concentrations of 12.5, 25 and 50 µM did not show any toxic effect on the growth of the THP-1 cells. Henceforth we have tested the mentioned concentrations of 25 and 50 µM for our following experiments.

### 2.2. Effect of BBR on Arachidonic Acid (AA)-Induced Proinflammatory Markers

**Figure 2:**
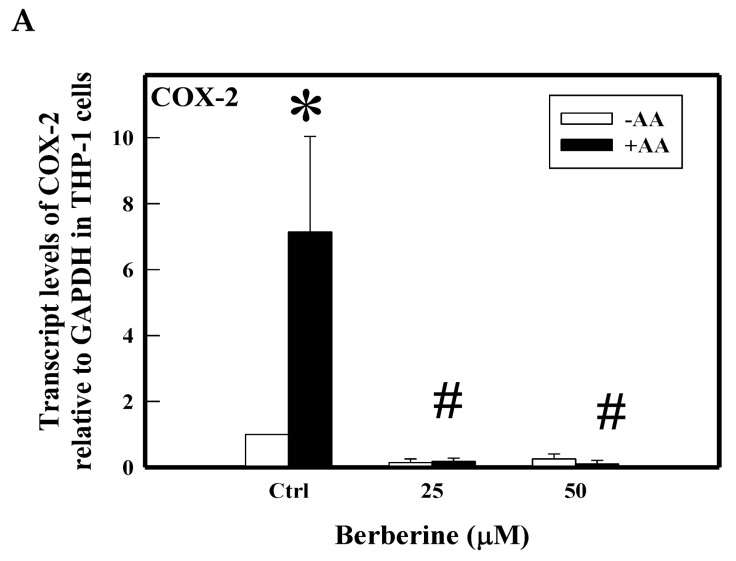

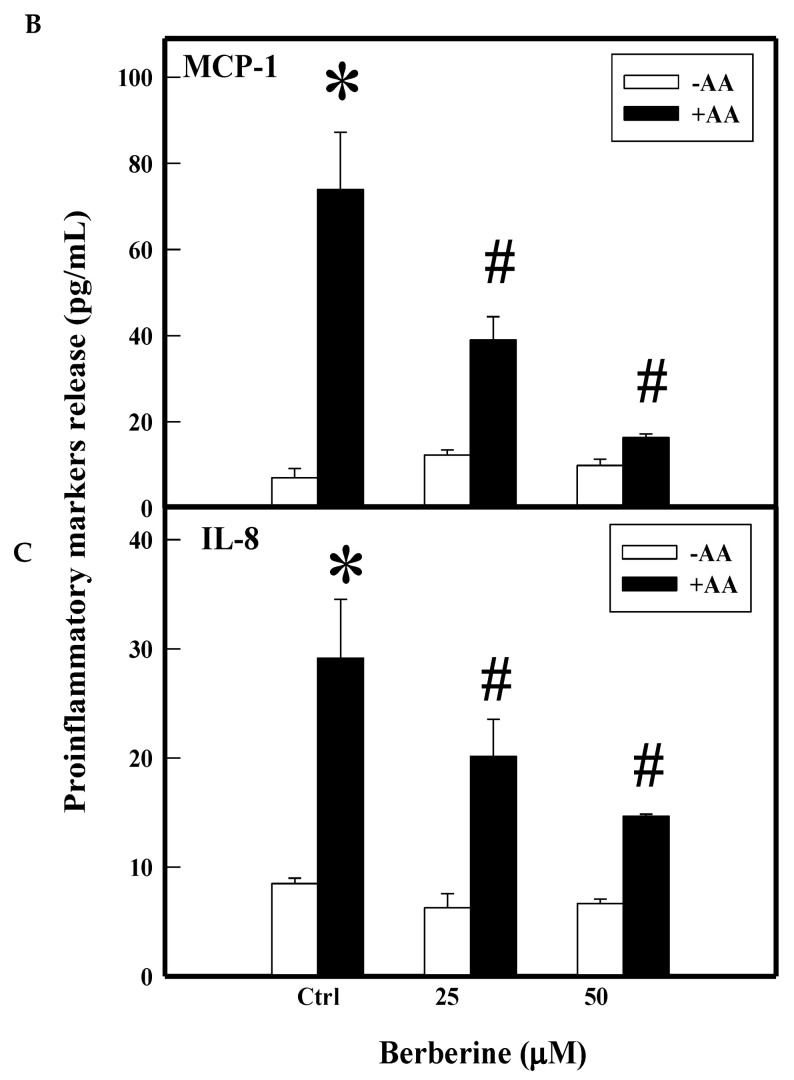
Effect of BBR on AA-induced COX-2 (**A**) gene transcript and proinflammatory cytokines. THP-1 cells were pretreated with BBR followed by induction with AA. At the end of the treatment, cells were centrifuged. Pelleted cells were subjected to RNA isolation and gene transcript of COX-2 assayed by RT-PCR and supernatants were assayed for proinflammatory cytokines MCP-1 (**B**) and IL-8 (**C**) by ELISA. Ctrl (control) represents cells incubated without berberine. Data represent mean ± SD of three independent experiments and # indicates *p* < 0.001 compared between treated and untreated with controls, * indicates *p* < 0.001 between the control groups.

As shown in earlier scientific reports [23], AA treatment induced proinflammatory markers in THP1 cells by several folds compared to control cells (Figure 2). We tested the inhibitory effect of BBR (25, 50 µM) on AA-induced COX-2 and inflammatory cytokines using real time PCR and ELISA. Results clearly show the significant attenuation of the above markers by BBR at 25 and 50 µM. COX-2 transcript was upregulated to 7-fold by arachidonic acid when compared to control cells, whereas pretreatment with BBR significantly attenuated such induction of COX-2 at both 25 µM and 50 µM (Figure 2A). Upon induction with AA, cells increased the release of MCP-1 (74 pg/mL) and IL-8 (29 pg/mL). Pretreatment of cells with BBR significantly inhibited AA-induced release of MCP-1 (39 and 16 pg/mL) and IL-8 (20 and 15 pg/mL) (Figure 2B,C).

### 2.3. Effect of BBR on LPS Induced Proinflammatory Markers

**Figure 3:**
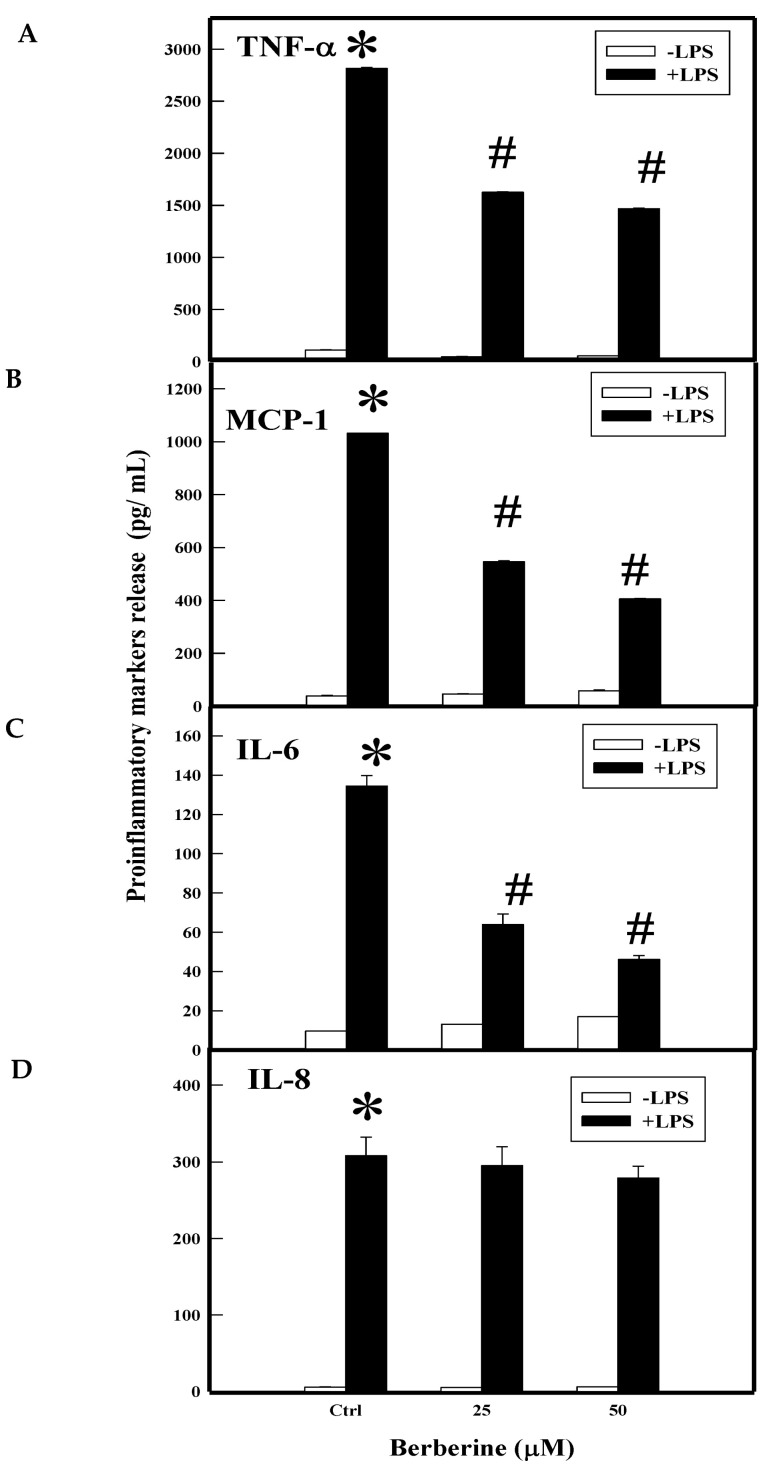
Effect of BBR on LPS-induced proinflammatory markers by ELISA. THP-1 cells were pretreated with BBR and induced with LPS. At the end of the treatment, cells were spun and the culture supernatant collected. Protein levels of the markers TNF-α (**A**), MCP-1 (**B**), IL-6 (**C**), and IL-8 (**D**) were quantified by ELISA. Ctrl (control) represents cells incubated without berberine. Data represent mean ± SD of three independent experiments and # indicates *p* < 0.001 compared between treated and untreated with controls, * indicates *p* < 0.001 between the control groups.

The activated monocytes secrete various proinflammatory cytokines and chemokines (e.g., TNF-α, IL-6, IL-8, and MCP-1) that play important roles in atherogenic lesions. LPS induced the secretion of TNF-α (2816 pg/mL), MCP-1 (1031 pg/Ml), IL-6 (134 pg/mL), and IL-8 (308 pg/mL) by several fold in THP-1 cells. Pretreatment with BBR (25 µM and 50 µM) significantly attenuated LPS-induced secretion of TNF-α (1625 and 1467 pg/mL), MCP-1 (546 and 405 pg/mL), and IL-6 (64 and 46 pg/mL) at 25 and 50 µM, respectively, but did not show much protection towards IL-8 (295 and 279 pg/mL) as shown in Figure 3.

**Figure 4:**
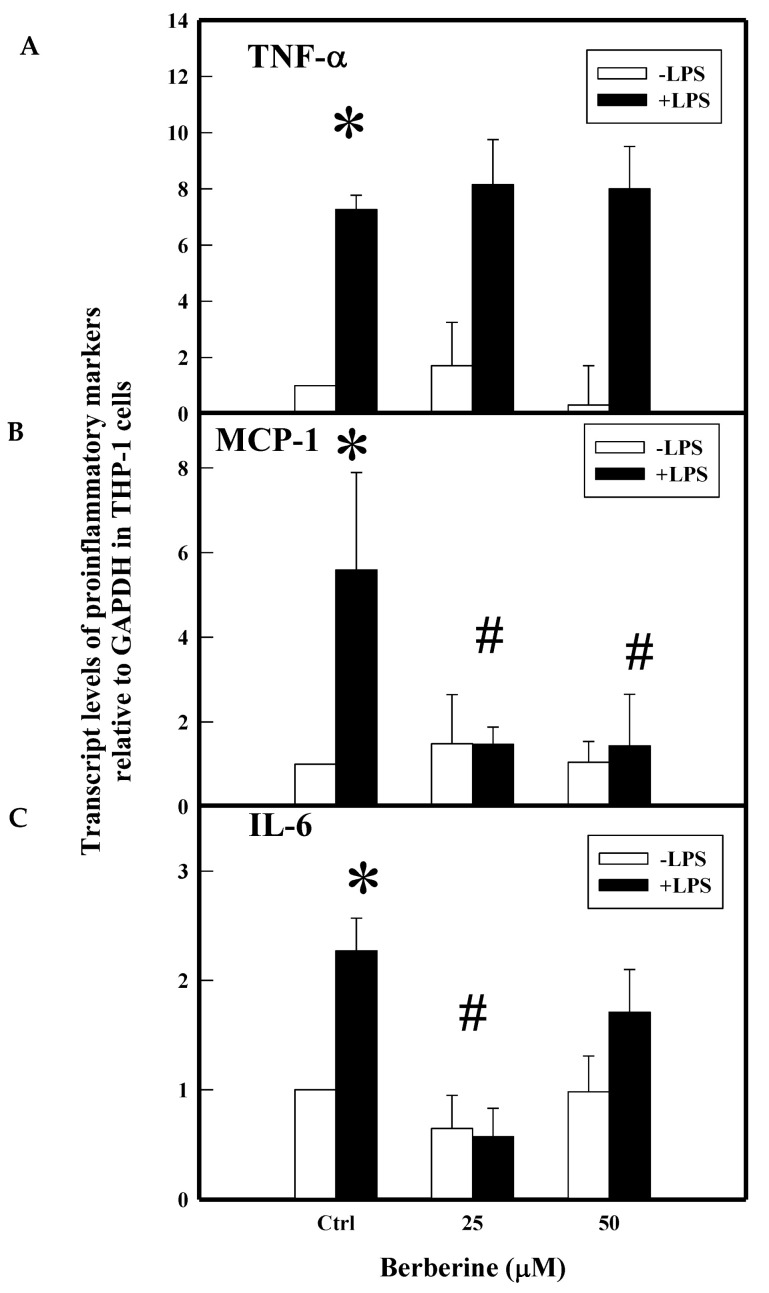
Effect of BBR on LPS-induced proinflammatory gene transcripts. THP-1 cells were pretreated with BBR and induced with LPS for 3 h. At the end of the treatment, RNA was isolated using TRIzol reagent. Gene transcripts (normalized with GAPDH) of TNF-α (**A**), MCP-1 (**B**), and IL-6 (**C**) were quantified by real time PCR. Ctrl (control) represents cells incubated without berberine. Data represent mean ± SD of three independent experiments and # indicates *p* < 0.001 compared between treated and untreated with controls, * indicates *p* < 0.001 between the control groups.

Transcriptional regulation is the control point for the secretion of these inflammatory cytokines at the protein level. We demonstrated the effect of BBR on m-RNA levels for these genes in THP-1 cells, stimulated in the presence or absence of LPS (0.5 µg/mL). Results in Figure 4 demonstrated the upregulation of TNF-α (7.3-fold), IL-6 (2.3-fold), and MCP-1 (5.6-fold) gene transcripts in LPS-treated cells when compared to normal control cells. Pretreatment with BBR at 25 and 50 µM concentrations significantly downregulated MCP-1 (1.5, 1.4-fold) and IL-6 (0.6, 1.7-fold) genes. However, BBR did not show such inhibitory effect on the induction of TNF-α (8 fold) transcript in THP-1 cells. This data may suggest that BBR carries out the inhibitory effect against TNF-α at the post-transcriptional level, which, however, needs to be investigated. The post-transcriptional inhibitory effect of BBR on TNF-α with an unknown molecular mechanism is hypothesized elsewhere [24]. 

### 2.4. Effect of BBR on LPS-Induced NF-κB p-65 Translocation in THP-1 Cells

**Figure 5:**
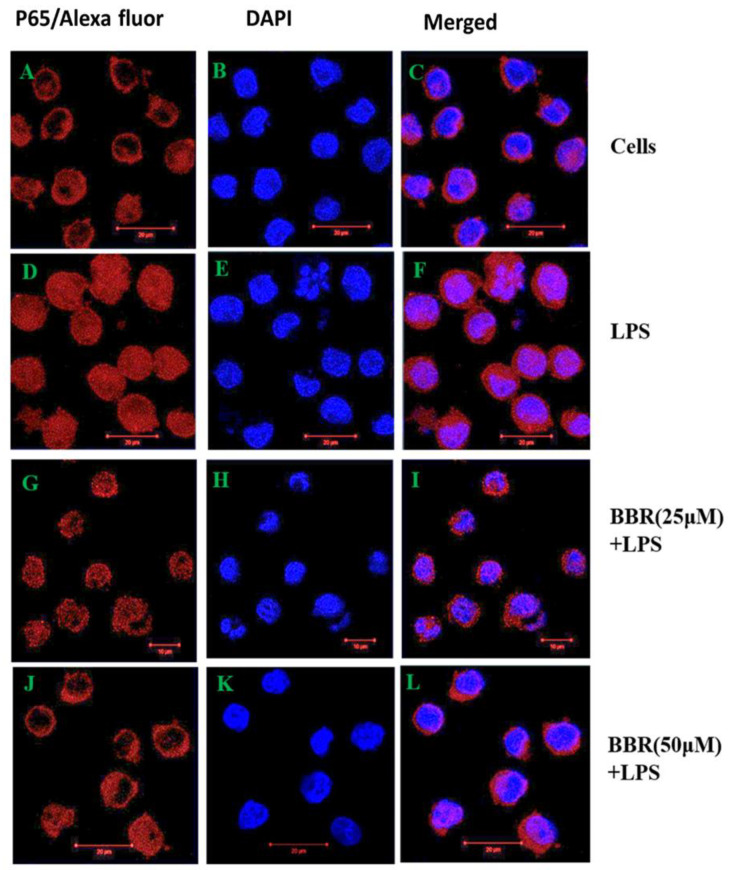
Effect of BBR on LPS-induced NF-κB P-65 subunit translocation. THP-1 cells were pretreated with BBR (25 and 50 μM) then induced with LPS (0.5 μg/mL), and the subcellular localisation of the p65 subunit was determined by the immunofluorescence technique. The NF-κB p65 subunit was stained with Alexa Fluor 594 conjugated secondary antibody and the nucleus was counterstained with DAPI. Images were captured under a confocal microscope (Zeiss, Germany). (**A**,**D**,**G**,**J**) are Alexa Fluor labelled cells; (**B**,**E**,**H**,**K**) are DAPI stained cells and (**C**,**F**,**I**,**L**) are merged images of Alexa Fluor and DAPI stained cells, respectively.

**Figure 6:**
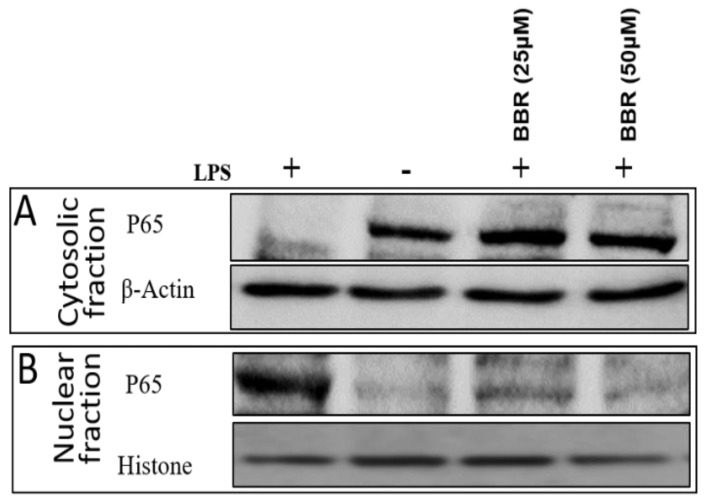
Effect of BBR on LPS-induced NF-κB P-65 subunit translocation. THP-1 cells were pretreated with BBR, then induced with LPS (0.5 μg/mL) and protein lysate of cytosol (**A**) and nuclear extracts (**B**) was extracted using NE-PER buffer. Lysates were loaded onto 10% SDS PAGE and transferred to blotting membrane, probed with primary antibody of NF-κB P-65. Finally, NF-κB levels were detected by ECL reagents using VersaDoc. For loading controls, antibodies against GAPDH (**A**) and histone (**B**) were used for cytosolic and nuclear fractions, respectively.

Transcripts of proinflammatory cytokines are known to be regulated by transcription factor NF-κB. To determine whether BBR attenuates pro-inflammatory marker expression through the suppression of nuclear translocation of NF-κB, we tested the effect of BBR on NF-κB targeting into the nucleus by immunofluorescence and Western blot methods and the results are presented in the Figure 5 and Figure 6. Immunofluorescence (Figure 5D–F) showed the stimulation of NF-κB translocation by LPS into the nucleus, unlike control cells, which retained the protein in the cytosol (Figure 5A–C). The pretreatment with BBR (25, 50 μM) significantly sequestered NF-κB in the cytosol, in the inactive state (Figure 5G–L), similar to uninduced cells.

Further, immunoblotting analysis showed that the THP-1 cells stimulated with LPS translocated the p65 subunit of heteromeric NF-κB protein into the nucleus in contrast to the uninduced cells, which retained the p65 protein in the cytosol. Pretreatment of LPS-stimulated cells with BBR attenuated the p65 translocation, which was confirmed by the presence of p65 bands in the cytosol and marginally reduced band in the nucleus. Cytosolic and nuclear fraction protein samples were confirmed by the presence of the marker proteins GAPDH and histone, respectively (Figure 6).

### 2.5. Molecular Modeling

**Figure 7:**
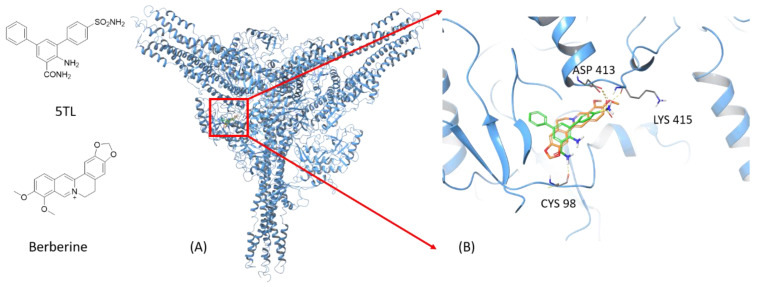
Docking results of BBR and 5TL to IKKα. Hydrogen bonds were interpreted as yellow dash lines. Carbons in 5TL were coloured green and those in BBR were coloured gold. All non-polar hydrogens were hidden. The structures of 5TL and BBR, the binding of both ligands (**A**) and the focused binding site of both ligands (**B**) are shown in the figure.

To preliminarily understand the molecular mechanisms leading to BBR’s anti-inflammatory activities, we performed molecular modeling studies. These modeling studies suggested the direct interactions between BBR to IKKα, using the co-crystal structure of IKKα in complex with its inhibitor 5TL. The best binding poses of 5TL and BBR are shown in Figure 7. 5TL and BBR have comparable docking scores (−7.1 kcal/mol versus −6.5 kcal/mol, respectively), although 5TL has more hydrogen bonds with IKKα than BBR does. The two molecules overlap at the ligand binding site well, with both molecules forming a strong hydrogen bond to Asp413 with additional hydrophobic interactions from the surrounding residues. While very preliminary, this modeling study suggests that BBR has comparable binding affinity with the known IKKα inhibitor 5TL and thus potentially interact with IKKα directly.

## 3. Discussion

Oxidative stress, caused due to the imbalance between ROS generation and scavenging during cellular metabolism, would result in pathophysiology by damaging lipids, proteins, and nucleic acids [25,26,27]. Arachidonic acid metabolism induces inflammation by secreting inflammatory mediators (TNF-α) via oxidative burst releasing ROS [22]. It is involved in the COX-2-mediated synthesis of eicosanoids, which are considered essential contributors to the pathogenesis of atherosclerosis. Eicosanoids activate and recruit monocytes to the site of inflammation and alter the gene expression pattern of many proinflammatory markers in vascular endothelial and smooth muscle cells, promoting inflammatory pathogenesis. In treating atherosclerosis, it is highly essential to protect monocytes from being exposed to COX-2 products i.e., eicosanoids. Our results demonstrated attenuation of AA-induced proinflammatory markers like MCP-1, IL-8 secretion, and COX-2 gene expression by BBR in THP-1 cells. 

Activation and adhesion of monocytes to endothelium are preliminary steps leading to atherosclerosis [28,29]. Inhibitors of such molecular interactions between the activated monocytes and HAECs can be considered to develop novel therapeutic candidates for many chronic inflammatory diseases. Previous studies of BBR have shown that this alkaloid can reduce LPS-induced lung injury by attenuating VCAM-1 in both in vitro (HUVECs) and in vivo (male Sprague Dawley rats) models. It is also reported that this alkaloid attenuated inflammatory and cell cycle regulated genes significantly in PMA differentiated THP- cells [30]. Literature strongly supports anti-inflammatory [24], anticoagulant [31], and antiatherogenic [32] activities of BBR. BBR inhibits LPS-induced tissue factor via NF-κB, AKT, and MAPK pathway in THP-1 cells [31]. BBR is also proved to attenuate foam cell formation in THP-1-derived macrophages by activating sirtuin 1 via AMPK pathway [32]. Further, BBR was reported to scavenge superoxide production mediated by NADPH oxidase in macrophages [33]. In an extension of these studies, we demonstrated the anti-inflammatory effect of BBR via NF-κB pathway THP-1 (monocytes) and possibly by inhibiting IKKα.

TNF-α is the most important cytokine secreted by the activated monocytes/macrophages, and it upregulates the expression of sticky adhesive proteins like VCAM-1, ICAM-1, and E-selectin present on the surface layer of the endothelium [24,34,35,36]. It acts as a prominent inflammatory mediator through the secretion of other cytokines such as IL-6, IL-8, and MCP-1 [37,38]. Among these secretory products, IL-6 and IL-8 have significant roles in inflammation. They cause the rolling of monocytes to firmly adhere to the endothelial monolayer expressing VCAM-1, ICAM-1, and E-selectin [37]. IL-8 is also mitogenic and chemotactic for vascular smooth muscle cells (SMCs). MCP-1, a chemokine released by monocytes and endothelial cells, promotes the transmigration and recruitment of monocytes and macrophages to the site of inflammation. Deposition of lipids within these cells leads to development of atherosclerotic lesions. In our study, BBR significantly attenuated LPS-induced TNF-α, MCP-1 and IL-8 secretion by THP-1 cells, showing its anti-inflammatory effect in monocytic cells. However, TNF-α is attenuated at post-transcription with an unknown molecular mechanism, as supported by the previous study in human subjects [24].

NF-κB is a pivot transcription factor, which controls the expression of many pro-inflammatory genes [39,40,41]. Stimulation of THP-1 cells with LPS translocates the p65 subunit of heteromeric NF-κB protein into the nucleus, by phosphorylation and degradation of the I-κB subunit, and turns on the expression of various proinflammatory genes by binding to their promoter regions. To understand the mechanism of action of BBR, we performed a docking study between BBR and IKKα, an upstream protein of NF-κB. The results showed a comparable binding affinity between BBR and IKKα to a known IKKα inhibitor 5TL. This suggests that BBR may inhibit the LPS-induced NF-κB translocation by inhibiting IKKα. Thus, our study unveiled the molecular mechanism of BBR in attenuating the above LPS-induced inflammatory genes, via NF-κB pathway through the sequestration of inactive p-65 subunit as revealed in our immunofluorescent and blotting studies. Our findings firstly reported the in vitro anti-inflammatory functions of BBR in activated THP-1 cells by attenuation of the nuclear translocation of p-65 in THP-1 cells which in turn support the interpretation that therapeutic properties of BBR can be exploited for the treatment of inflammatory diseases like atherosclerosis. However, the current data are not sufficient to explain the beneficial effects of BBR in in vivo systems. Therefore, further studies in this area would be needed.

## 4. Materials and Methods

### 4.1. Chemicals

Arachidonic acid (AA), berberine (BBR), Trypan Blue, lipopolysaccharide—*E. coli* (LPS), and DEPC-treated water were obtained from Sigma–Aldrich (Saint Louis, MI, USA). RPMI 1640 medium, L-glutamine, PenStrep, fetal bovine serum (FBS), Alexa Fluor 594, goat anti-rabbit IgG, prolonged gold anti-fade reagent with 4’,6-diamidino-2-phenylindole (DAPI), and Trizol reagent were obtained from Invitrogen (Erie County, New York, United States). 3-(4,5-dimethylthiazol-2-yl)-2,5-diphenyltetrazolium bromide (MTT) was obtained from Himedia (Mumbai, Maharashtra, India). ELISA kits were procured from BD Biosciences (San Diego, CA, USA) with catalogue numbers TNF-a (555212), MCP-1 (555179); IL-8 (555244) and il-6 (555220). iScript cDNA synthesis kit was purchased from Bio-Rad (Hercules, CA, USA), and Power SYBR Green PCR master mix was obtained from Applied Biosystems (Carlsbad, CA, USA). NF-κB p65 subunit polyclonal rabbit antibody was obtained from Thermo Scientific (Rockford, IL, USA). All other reagents used were of analytical grade.

### 4.2. Cell culture Treatments

Human monocytic (THP-1) cell line was obtained from National Centre for Cell Science (NCCS Pune, India) and cultured as described previously [42]. Cells (0.5 × 10^6^) were treated with various concentrations of BBR (12.5, 25 and 50 µM) for 16 hrs and cell viability was performed by Trypan blue exclusion method as well as by MTT assay (Figure 1) as described elsewhere [42,43,44]. Cells were subjected to inflammatory stimulus by treating with LPS 0.5 µg/mL for 3 h, 6 h and 12 h or with AA (100 µM) for 24 h at 37 ℃ and 5% CO_2_.

### 4.3. Measurement of Cytokines and Chemokines

THP-1 cells at a density of 0.5 × 10^6^ cells/mL were induced with LPS (0.5 µg/mL) for 3 h (TNF-α, IL-8), 6 h (MCP-1) or 12 h (IL-6) or AA (100 µM) for 24 h. Cell culture supernatants collected by centrifugation were subjected to quantification of the secreted proinflammatory cytokines and chemokines such as TNF-α, IL-6, IL-8, and MCP-1 using commercially available ELISA kits (BD Biosciences, San Jose, CA, USA) according to manufacturer’s instructions. Cells treated with BBR alone were used as controls.

### 4.4. Quantitative Real-Time PCR Analysis

Total RNA was isolated from the THP-1 cells by using TRIzol reagent according to manufacturer’s protocol. RNA was converted to cDNA using iScript^TM^ cDNA synthesis kit. Quantitative real -time PCR analysis was performed by 1x SYBER green PCR reagent using primers specific for proinflammatory genes [45,46,47]. Glyceraldehyde-3-phosphate dehydrogenase (GAPDH) with real time sequences (forward primer 5′-CACCAACTGCTTAGCACCCC-3′; reverse primer 5′-TGGTCATGAGTCCTTCCACG-3′) is used as an internal control housekeeping gene to normalize gene expression using 2^−ΔΔCt^ formula. The primer sequences for the tested genes (Eurofins genomics, Bangalore, India) are given in the Table 1.

### 4.5. Immunofluorescence Staining

After the appropriate treatments, THP-1 cells were fixed with 4% paraformaldehyde for 15 min and permeabilized with 0.5% Triton X-100 for 15 min. After 1 h blocking with BSA, cells were incubated with rabbit anti-NF-κB p65 for 2 h, washed and then incubated with diluted Alexa Fluor dye for 1 h [48]. To identify the nuclei, samples were stained with DAPI for 15 min and the fluorescent images were finally captured using a confocal laser scanning electron microscope (Zeiss, Jena, Germany).

### 4.6. Immunoblotting

THP-1 cells were treated with BBR overnight followed by LPS treatment for 1 h. After the treatments, cells were lysed in a nuclear extraction (NE-PER) buffer in order to obtain cytosol and nuclear fractions as per manufacturer’s protocol. The total protein in both fractions was measured by Bradford’s method. Cytosolic and nuclear protein fractions (50 μg/lane) were loaded and subjected to SDS-PAGE and then wet transferred onto nitrocellulose membrane (NCM). The membrane was blocked with 15% milk powder in TBS at room temperature for 1 h and then probed with primary anti.rabbit NFp65 antibody (1:1000) overnight at 4 ℃. After incubation, NCM was washed with TBST for 1 h and incubated with anti-rabbit HRP secondary antibody (1:3000). Antibodies of GAPDH (1:1000) or histone (1:1000) were used as internal references for the equal loading of cytosol or nuclear extracts, respectively. Finally, an enhanced ECL reagent was used for blot visualization in a Bio-Rad chemidoc imaging system (USA) using chemidoc imaging software (Bio-Rad, Hercules, CA, USA)

### 4.7. Molecular Modeling

The protein-ligand docking studies were performed based on the cocrystal structure of human inhibitor of nuclear factor kappa-B (IκB) kinase subunit alpha (IKKα) and 5TL, a known inhibitor of IKKα [49] [PDB: 5EBZ [50]. The Schrodinger Molecular Modeling Suite 2020 was used for this modeling study (Schrodinger, Inc., New York, NY, USA), similar to what we have done in previous studies [PMID: 29406710; PMID: 22783954]. One hexamer was used for the preparation by the protein preparation wizard module in Maestro, during which bond orders were assigned, hydrogens were added to the PDB structure and the ionization state in pH 7.0 ± 2.0 was generated using the Epik module. The structures of 5TL and BBR were prepared by the Ligprep module with the OPLS3e force field. The receptor grid was generated using the Glide module. The sulfhydryl group in Cys98 and the hydroxyl group in Asp413 were allowed to rotate in order to sample their optimal orientations. Docking of 5TL and BBR were performed by ligand docking in the Glide module with standard precision followed by post-docking optimization [PMID: 29406710; PMID: 22783954].

### 4.8. Statistical Analysis

The entire data were expressed as mean ± standard deviation (SD) for triplicates and a statistical analysis was performed by one-way analysis of variance (ANOVA) test using SPSS 11 statistical software. # indicates *p* < 0.001 compared between treated and untreated with controls, * indicates *p* < 0.001 between the control groups.

## 5. Conclusions

These findings support BBR as a potential drug candidate for treating atherosclerosis, a chronic inflammatory blood vessel wall disease, through its anti-inflammatory activities. BBR exhibits anti-inflammatory activity in activated THP-1 by targeting NF-κB translocation. This study suggests that BBR could be an alternative drug to treat various inflammatory diseases, especially atherosclerosis.

## Figures and Tables

**Table 1:** Primers used for real time quantitative PCR assays.

Genes	Primer Sequences	Direction of the PCR Primer
TNF-α	5′-CCCAGGGACCTCTCTCTAATC-3′	Forward
	5′-ATGGGCTACAGGCTTGTCACT-3′	Reverse
MCP-1	5′GCCAAGGAGATCTGTGCTGAC-3′	Forward
	5′-CATGGAATCCTGAACCCACTTC-3′	Reverse
IL-6	5′-TGGATTCAATGAGGAGACTTGC-3′	Forward
	5′-CAGGAACTGGATCAGGACTT-3′	Reverse
IL-8	5′-GTGTAAACATGACTTCCAAGCTGG-3′	Forward
	5′-GCACCTTCACACAGAGCTGC-3′	Reverse
COX-2	5′-CAGCACTTCACGCATCAGTT-3′	Forward
	5′-CGCAGTTTACGCTGTCTAGC-3′	Reverse

## Sample Availability

Samples of the compounds are not available from the authors.
